# Clinical validation of a blood-based classifier for diagnostic evaluation of asymptomatic individuals with pulmonary nodules

**DOI:** 10.1186/s12014-017-9158-9

**Published:** 2017-07-05

**Authors:** Charles E. Birse, Jennifer L. Tomic, Harvey I. Pass, William N. Rom, Robert J. Lagier

**Affiliations:** 1 Quest Diagnostics, Research and Development, 33608 Ortega Highway, San Juan Capistrano, CA 92675 USA; 2 Department of Cardiothoracic Surgery, NYU Langone Medical Center, 530 First Avenue, New York, NY 10016 USA; 3 Division of Pulmonary, Critical Care, and Sleep Medicine, NYU School of Medicine, New York, NY 10016 USA; 4 Grifols Diagnostic Solutions, 4560 Horton St., Emeryville, CA 94608 USA

**Keywords:** Lung cancer screening, Indeterminate pulmonary nodules, Biomarker, Serum, Asymptomatic

## Abstract

**Background:**

The number of pulmonary nodules detected in the US is expected to increase substantially following recent recommendations for nationwide CT-based lung cancer screening. Given the low specificity of CT screening, non-invasive adjuvant methods are needed to differentiate cancerous lesions from benign nodules to help avoid unnecessary invasive procedures in the asymptomatic population. We have constructed a serum-based multi-biomarker panel and assessed its clinical accuracy in a retrospective analysis of samples collected from participants with suspicious radiographic findings in the Prostate, Lung, Chest and Ovarian (PLCO) cancer screening trial.

**Methods:**

Starting with a set of 9 candidate biomarkers, we identified 8 that exhibited limited pre-analytical variability with increasing clotting time, a key pre-analytical variable associated with the collection of serum. These 8 biomarkers were evaluated in a training study consisting of 95 stage I NSCLC patients and 186 smoker controls where a 5-biomarker pulmonary nodule classifier (PNC) was selected. The clinical accuracy of the PNC was determined in a blinded study of asymptomatic individuals comprising 119 confirmed malignant nodule cases and 119 benign nodule controls selected from the PLCO screening trial.

**Results:**

A PNC comprising 5 biomarkers: CEA, CYFRA 21-1, OPN, SCC, and TFPI, was selected in the training study. In an independent validation study, the PNC resolved lung cancer cases from benign nodule controls with an AUC of 0.653 (p < 0.0001). CEA and CYFRA 21-1, two of the markers included in the PNC, also accurately distinguished malignant lesions from benign controls.

**Conclusions:**

A 5-biomarker blood test has been developed for the diagnostic evaluation of asymptomatic individuals with solitary pulmonary nodules.

**Electronic supplementary material:**

The online version of this article (doi:10.1186/s12014-017-9158-9) contains supplementary material, which is available to authorized users.

## Background

Pulmonary nodules are a common finding in routine clinical practice with an estimated 1.5 million new cases being detected annually in the United States [1]. Incidence is likely to increase in the coming years following the recommendations from the US Preventative Services Task Force (USPSTF) [2] and the Centers for Medicare & Medicaid Services (CMS) [3] for annual CT-based screening of the population with a high risk for lung cancer. Currently there are approximately 7 million current or former smokers in the US who meet the National Lung Screening Trial (NLST) criteria for screening. In the NLST, 24.2% of CT screening tests had a positive finding, with 96.4% of these initial findings representing false positives for lung cancer [4].

Given the poor prognosis associated with advanced stage disease, the goal in managing individuals with pulmonary nodules is to rapidly identify and resect malignant lesions while avoiding unnecessary invasive procedures in patients with benign disease. Guidelines for the management of pulmonary nodules have recently been revised by several professional organizations [5–7]. The American College of Radiology has initiated efforts to standardize reporting of results from low dose chest CT screening with the introduction of the Lung-RADS system, which emulates the Bi-RADS scoring system for mammography. Lung-RADS, which defines a positive screening test and provides recommendations for management based on level of risk, has been adopted at many academic medical centers in the US [8]. The Lung-RADS system increases the size threshold for a positive baseline finding from 4 mm, used in the NLST, to 6 mm. ≥6 to <8 mm nodules (Category 3) are considered “probably benign”, with a risk of malignancy of 1–2% and should be managed with a follow-up low dose CT scan in 6 months. Category 4 nodules are considered “suspicious”: ≥8 to <15 mm nodules (Category 4A) have a 5–15% cancer risk and should be managed with a 3 month LDCT scan, or PET/CT may be used if there is a ≥8 mm solid component; >15 mm nodules (Category 4B) have a cancer risk ≥15% and should be managed with a chest CT with/without contrast, PET/CT and/or biopsy, depending on the risk of malignancy and comorbidities. Despite these updated recommendations, invasive procedures are still performed on 44% of subjects with low-risk nodules (<5% probability of malignancy), and 35% of surgical resections are ultimately determined to be benign disease [9].

Integration of blood-based biomarkers may increase the accuracy of nodule classification, improving diagnostic evaluation, and thereby reducing the number of unnecessary and costly invasive procedures [10]. Although some promising candidate protein molecules have been evaluated as single markers [11, 12], given the heterogeneity of non-small cell lung cancers, it seems likely that multi-marker panels will be required to provide sufficient test sensitivity to make a meaningful clinical impact in nodule management. Some multi-marker adjunctive tests have been shown to perform with modest diagnostic accuracy in distinguishing benign from malignant lesions in subjects with indeterminate pulmonary nodules [13–18].

Given the recent recommendations from USPSTF and CMS for CT-based screening of those at high risk of lung cancer, there is a pressing need to investigate the performance of proteomic biomarkers in this asymptomatic population. While a number of well characterized markers including CEA, CYFRA 21-1 and SCC have previously been assessed for a wide range of diagnostic, prognostic and monitoring applications, they have not been evaluated as adjunctive biomarkers in the radiographic screening of asymptomatic individuals for lung cancer [19–22]. Some investigations have assessed the performance of these markers in individuals with pulmonary nodules, but these studies, including earlier work in our laboratory [23], have included symptomatic lung cancer cases, or controls not restricted to subjects with benign nodules [13, 14, 24].

We previously identified a diverse repertoire of candidate lung cancer biomarkers using a mass spectrometry-based discovery approach. We now report on the transfer of these markers onto a multiplex Luminex platform, thereby increasing throughput and reducing sample volume requirements. Using this multiplex assay, we deselected biomarkers affected by pre-analytical variables associated with blood collection and processing [25–28]. Thereafter, we developed a serum-based pulmonary nodule classifier (PNC) comprising 5 biomarkers: CEA, CYFRA 21-1, OPN, SCC, and TFPI. We then determined the clinical accuracy of the PNC, together with the performance of the individual contributing biomarkers, in asymptomatic subjects with suspicious radiographic abnormalities from the Prostate, Lung, Chest and Ovarian (PLCO) cancer screening trial [29]. We believe this study provides the first evaluation of clinical accuracy of these biomarkers in individuals with solitary pulmonary nodules resolved through radiographic screening for lung cancer.

## Methods

### Pre-analytical variability study

Blood was drawn from healthy volunteers (n = 6). From each individual, 3 blood samples were collected into red-top serum tubes (Becton Dickinson, Franklin Lakes, NJ, #367820) and blood was allowed to clot at room temperature for: 0.5, 4, or 24 h. After clotting, tubes were spun at 1200*g* for 10 min at room temperature. 2 mL aliquots of separated serum were then transferred to −80 °C for long-term storage.

### Training study (New York University, Clinical Research Center at Cape Cod)

Serum was collected from healthy smoker controls (n = 186) and patients with early-stage non-small-cell lung cancer (NSCLC; n = 95) (Table 1).

**Table 1:** Demographics of the training and validation study populations

	Training		Validation	
	Control (n = 186)	NSCLC (n = 95)	CXR finding: nodule	
			Control (n = 119)	Lung cancer (n = 119)
Gender				
Male	107	34	69	69
Female	79	61	50	50
Age (years)				
Mean (SD)	62.3 (11.8)	66.5 (9.6)	63.7 (5.2)	64.4 (5.3)
Smoking				
Ever smokers	186	95	106	106
Never smokers	–	–	13	13
Pack years, mean (SD)	37.4 (21.5)	44.5 (21.3)	46.0 (32.4)	55.3 (40.9)
Abnormal suspicious finding				
Nodule	NA	NA	119	119
Stage				
I		95 (100%)		5 (4.2%)
Ia		–		51 (42.9%)
Ib		–		15 (12.6%)
II		–		10 (8.4%)
III		–		19 (16.0%)
IV		–		14 (11.8%)
NA		–		5 (4.2%)
Histology				
Adenocarcinoma		63 (66.3%)		57 (47.9%)
BAC		4 (4.2%)		20 (16.8%)
Squamous cell		15 (15.8%)		25 (21.0%)
Large cell		6 (6.3%)		4 (3.4%)
NSCLC		4 (4.2%)		2 (1.7%)
Neuroendocrine		2 (2.1%)		2 (1.7%)
Small cell		–		5 (4.2%)
Adenosquamous		1 (1.1%)		4 (3.4%)

The design and subjects evaluated, are very similar to a previous training study where biomarker levels were evaluated using an ELISA platform [23].

### Validation study (PLCO)

PLCO biospecimens were collected and processed using uniform procedures. Samples were collected prospectively (pre-diagnosis), eliminating any inherent case–control bias [30].

In the intervention (screening) arm of the PLCO study, 77,445 participants received a chest radiograph at baseline, then annually for 2 or 3 years [31]. Chest radiographs were considered positive if a nodule, mass, or other suspicious abnormality (atelectasis, pleural, hilar or mediastinal mass) was found. The prevalence of screen-detect cancers was strongly influenced by the type of abnormality. The PPV for a nodule was 1.3% (170/13,449), for a mass 8.7% (105/1208), and for other suspicious abnormalities 1.9% (63/3356) [32, 33]. Cancers were staged based on the fifth edition of the American Joint Committee on Cancer Staging Manual [34].

Subjects with solitary abnormal suspicious findings (nodule, mass, other) were considered for our validation study. Cases selected included individuals with a nodule (<3 cm; n = 119), a mass (>3 cm; n = 50), or “other” findings (pleural mass, hilar, infiltrate; n = 28), for whom serum had been collected up to 12 months before the diagnosis of malignancy. Controls were required to have at least 2 years of follow-up. Controls were individually matched to cases based on: finding (nodule, mass, other), gender, age at randomization and smoking history (Tables 1, 2). 152 of the 222 cases (68%) included in the study met the NLST high risk criteria where participants were selected based on age (55–74 years) and smoking history (at least 30+ pack years).

**Table 2:** Demographics of the validation study populations (mass and other)

	Validation			
	CXR finding: mass (n = 100)		CXR finding: other (n = 56)	
	Control	Lung cancer	Control	Lung cancer
Gender				
Male	28	28	22	22
Female	22	22	6	6
Age (years)				
Mean (SD)	64.0 (4.9)	63.9 (4.9)	65.3 (5.5)	65.1 (4.9)
Smoking				
Ever smokers	45	45	28	28
Never smokers	5	5	–	–
Pack years mean (SD)	50.6 (35.1)	58.2 (37.9)	54.8 (32.9)	52.3 (30.9)
Abnormal suspicious finding				
Mass	50	50	–	–
Pleural Mass	–	–	2	2
Hilar	–	–	9	9
Infiltrate	–	–	17	17
Stage				
I (%)		16 (32.0)		9 (32.1)
II (%)		4 (8.0)		3 (10.7)
III (%)		16 (32.0)		3 (10.7)
IV (%)		10 (20.0)		8 (28.6)
NA		4 (8.0)		5 (17.9)
Histology				
Adenocarcinoma (%)		24 (48.0)		12 (42.9)
BAC (%)		3 (6.0)		2 (7.1)
Squamous cell (%)		9 (18.0)		7 (25.0)
Large cell (%)		4 (8.0)		1 (3.6)
NSCLC (%)		6 (12.0)		1 (3.6)
Small cell (%)		4 (8.0)		5 (17.9)

### Multiplex bead-based immunoassay

xMAP™ bead-based technology (Luminex Corp., Austin, TX) enables simultaneous analysis of multiple analytes in a single sample. To ensure measurement of linear responses for all 9 candidate lung cancer biomarkers over a broad dynamic range, 5 of the biomarkers (MMP2, OPN, SLPI, TFPI, and TIMP1) were measured in serum diluted ten-fold in assay buffer (#RD-48; R&D Systems/Bio-Techne, Minneapolis, MN). Levels of the 4 remaining biomarkers (CYFRA 21-1, CEA, SCC and MDK) were measured in undiluted serum. Biomarker-specific reagents employed in these studies are described in Additional file 1: Table S1. Primary antibodies were coupled to magnetic carboxylated beads (MagPlex^®^ Microspheres, Luminex Corp.), following the manufacturer’s procedure, with the addition of an ethanolamine quenching step prior to blocking. Secondary antibodies were labeled through EZ-Link Sulfo-NHS-Biotinylation (Thermo Scientific Pierce, Grand Island, NY).

xMAP™ assays were performed in 96-well format following the manufacturer’s protocol (Luminex Corp), with some modifications. 6 µL of standards or serum were transferred in triplicate to the plate. Conjugated bead mixtures were sonicated briefly before being added (24 µL) to each well. Plates were covered with foil tape, and shaken overnight at 4 °C. Following transfer to a pre-wetted filter plate (AcroPrep 96; Pall Corp, Port Washington, NY), wells were washed 3 times (#WA126; R&D Systems/Bio-Techne) using a 406EL washer (BioTek, Winooski, VT). Biotin-labeled secondary antibody (25 µL) was added to each well, and the plates were shaken at room temperature for 120 min. After washing, 25 µL of diluted Streptavidin-PE (# PJRS20; Prozyme, Hayward, CA) was added to each well, and the plate was shaken for 30 min. After another wash step, beads were resuspended in 100 µL of PBS before being read (Luminex 200). MasterPlex QT curve-fitting software (Hitachi Solutions America, San Bruno, CA) was used to analyze Luminex xMAP™ data.

### Statistical methods

The final set of biomarkers for the PNC was selected via elasticnet regularized logistic regression modeling of lung cancer status [35]. The elasticnet regularization (95% lasso, 5% ridge) constrains the regression coefficients in an effort to increase prediction accuracy by controlling for overfitting of the model to the training data. Additionally, elasticnet performs marker selection by forcing the coefficients of the least contributory markers to shrink to zero as the penalty parameter is increased. Stratified bootstrap resampling (10,000 iterations) was used to select the optimal regularization penalty. Subjects not selected in each bootstrap sample were scored as a test set by each penalized model. The regularization penalty which achieved the greatest mean AUC for the 10,000 test sets was selected for regularizing the final fit to the full training data set. We chose maximal mean AUC as the criterion for selection of the elasticnet penalty parameter as it is an accepted and commonly used measure of overall performance in the classification setting. Other measures such as NPV and PPV could have been used to select the penalty, but this would introduce a level of subjectivity into the method as one would need to determine appropriate levels of each of these performance measures without any clear guidelines for such decisions. Confidence intervals for AUCs and tests for differences in AUCs for paired samples were calculated by the DeLong [36] method. All statistical analyses were performed in R [37].

## Results

Previous evaluation of the 9 candidate lung cancer biomarkers was performed using singleplex assays configured using ELISA methodology [23]. A new multiplex version of the assay was developed to streamline clinical validation studies using Luminex technology. The multiplex assay is highly specific with <1% cross-reactivity among markers (Additional file 1: Table S2).

**Fig. 1:**
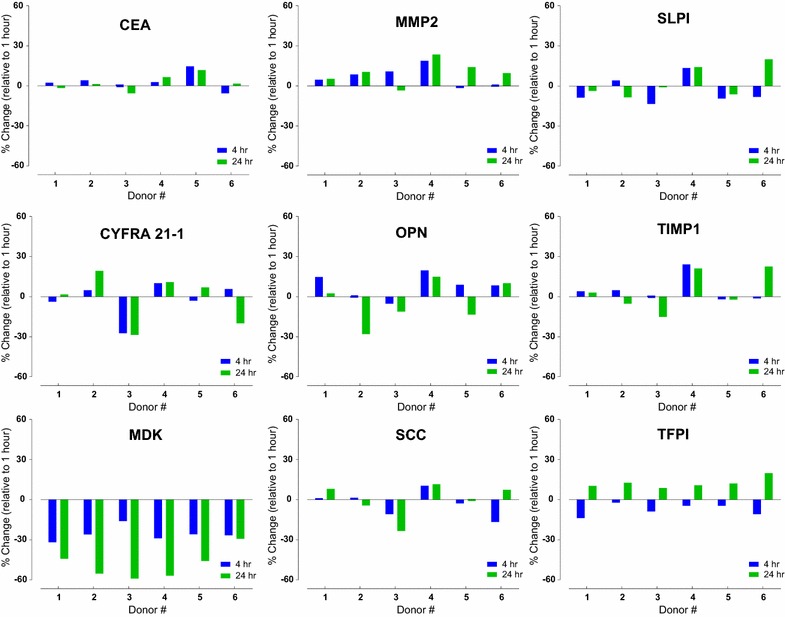
The effect of clotting time on marker levels in serum. Serum was collected from healthy donors (n = 6) after varying the blood clotting period: 0.5, 4, 24 h. Changes in marker levels observed after clotting for 4 h (*blue bar*) or 24 h (*green bar*) were plotted relative to the 0.5 h time point

One of the key pre-analytical variables in serum collection is clotting time, the period from venipuncture to centrifugation of the blood sample, with investigators employing a diverse spectrum of clotting-times, ranging from 30–60 min [38] to 24–56 h [27]. We evaluated the influence of clotting time in order to deselect candidate biomarkers affected by this key step in the pre-analytical processing pathway. We investigated the effect of clotting time on the serum levels of the 9 candidate lung cancer biomarkers, determining levels in six healthy individuals after clot formation for: 0.5, 4 or 24 h. The levels of most of the biomarkers showed only minor fluctuations at either 4 or 24 h, relative to the 0.5 h time point. However, levels of MDK fell consistently at both the 4-h (mean decrease 26%) and 24-h time points (mean decrease 48%), with lower levels always observed after 24 h (Fig. 1). Therefore, we excluded this biomarker from further evaluation.

Multi-marker algorithms started with the 8 biomarkers that showed minimal levels of pre-analytical variability. A 5-biomarker model (CEA, CYFRA 21-1, OPN, SCC and TFPI), a pulmonary nodule classifier (PNC), was selected from the training dataset (Additional file 1: Table S3). The PNC resolved malignant cases from healthy smoker controls with an AUC of 0.897 (95% CI 0.857–0.937; Additional file 2: Figure S1). A bootstrap validation procedure (10,000 iterations) confirmed the accuracy of the PNC in the training study: AUC was 0.894 (95% CI 0.822–0.944).

The accuracy of the PNC in distinguishing individuals with benign pulmonary nodules (n = 119) from those with malignant lesions (n = 119) was tested in serum collected pre-diagnosis from individuals participating in the PLCO cancer screening trial (Table 1). The PNC resolved the 2 populations with an AUC of 0.653, p < 0.0001 (Additional file 2: Figure S2). The performance of the individual biomarkers that constitute the classifier was also assessed, with CEA (AUC = 0.642, p < 0.0001) and CYFRA 21-1 (AUC = 0.628, p = 0.0004) accurately distinguishing cases from controls (Table 3).

**Table 3:** Diagnostic accuracy of pulmonary nodule classifier (PNC) and individual markers evaluated in the nodule population of the validation study

	Validation	
	CXR finding: nodule Benign control (n = 119); Lung cancer (n = 119)	
	AUC [95% CI]	p value
PNC	0.653 [0.583–0.723]	<0.0001
Individual markers included in PNC		
CEA	0.642 [0.572–0.713]	<0.0001
CYFRA 21-1	0.628 [0.558–0.699]	0.0004
SCC	0.567 [0.494–0.640]	0.0737
OPN	0.535 [0.461–0.609]	0.3508
TFPI	0.533 [0.459–0.606]	0.3815

**Fig. 2:**
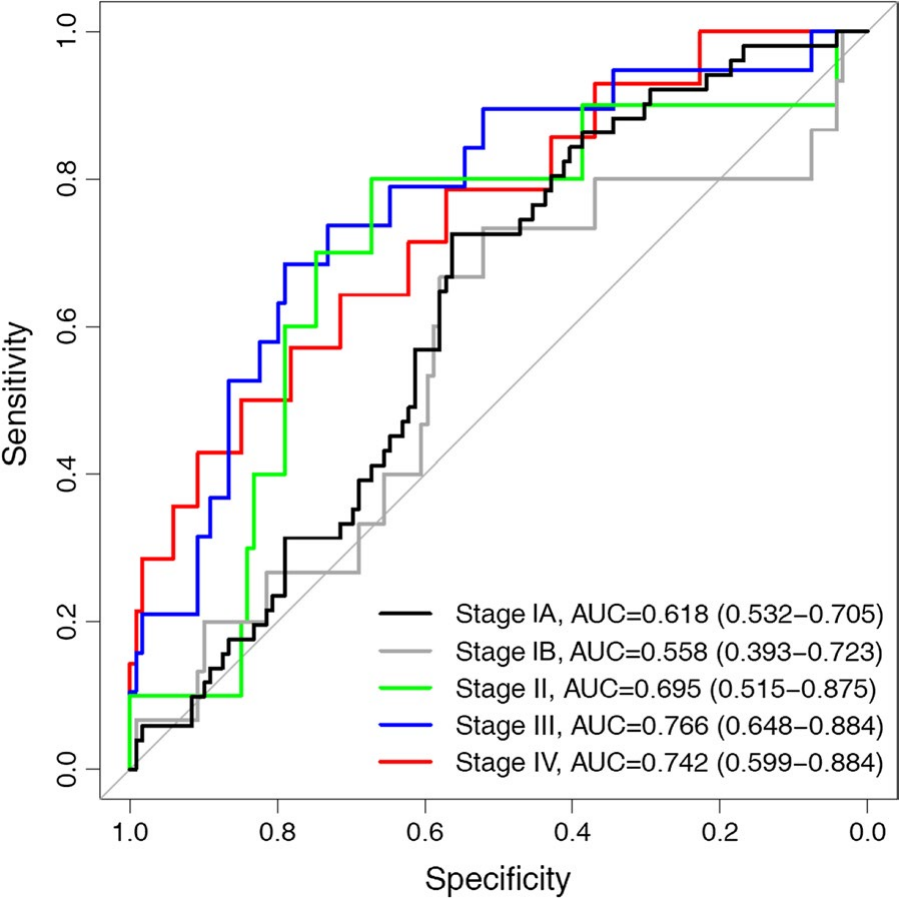
ROC curves showing diagnostic accuracy of pulmonary nodule classifier (PNC) evaluated in PLCO nodule population relative to tumor stage: stage IA (n = 51, *black*), stage IB (n = 15, *grey*), stage II (n = 10, *green*), stage III (n = 19, *blue*) and stage IV (n = 13, *red*). Area under the curve (AUC) and 95% confidence intervals are shown

The accuracy of the PNC in patients with pulmonary nodules was further characterized relative to tumor stage and histology. The multi-biomarker test showed modest performance in resolving stage IA cases (n = 51), which comprise 43% of the malignant nodules (AUC = 0.618, p = 0.0071; Fig. 2). Stage IA cases represent tumors ≤3 cm without bronchoscopic evidence of invasion more proximal than the lobar bronchus. Improved test accuracy was observed for cases diagnosed at later stages: stage II: AUC = 0.695 (p = 0.0341), stage III: AUC = 0.766 (p < 0.0001) and stage IV: AUC = 0.742 (p = 0.0009). Enhanced accuracy in resolving late stage disease (relative to stage I) was also noted for two biomarkers in the model: CEA and CYFRA 21-1 (Additional file 1: Table S4), as has previously been reported [19–22].

**Fig. 3:**
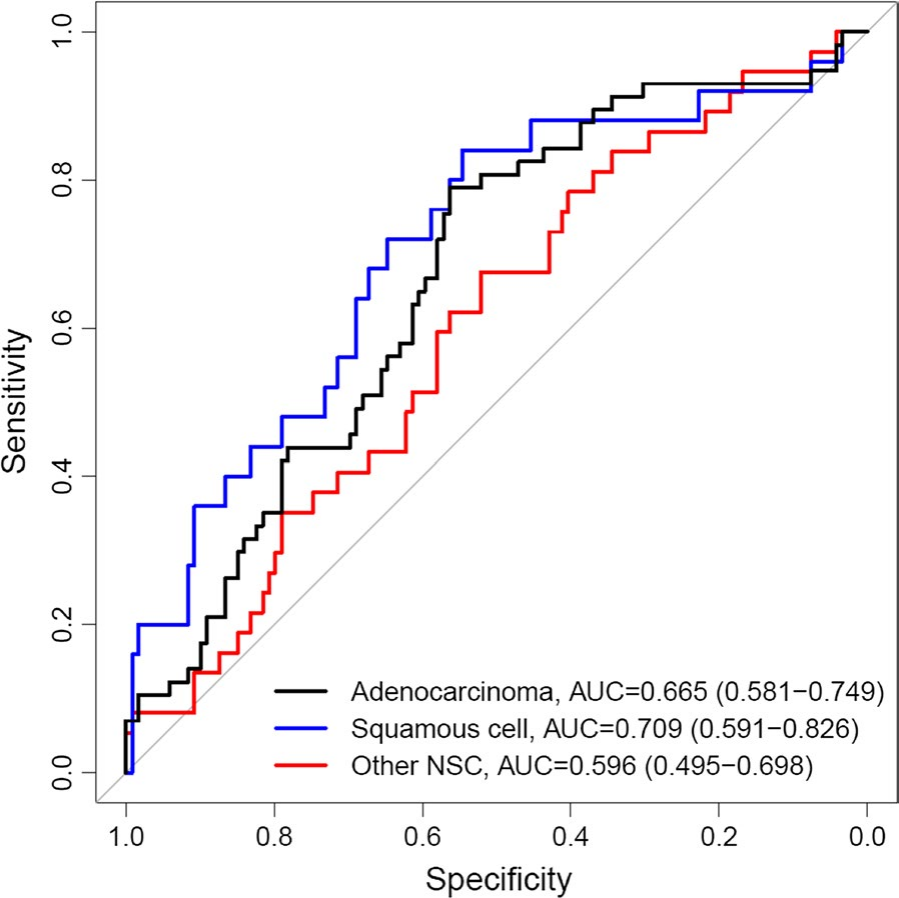
ROC curves showing diagnostic accuracy of pulmonary nodule classifier (PNC) evaluated in PLCO nodule population (n = 119) relative to tumor histology: adenocarcinoma (n = 57, *black*), squamous cell carcinoma (n = 25, *blue*), other non-small cell carcinomas (n = 37, *red*). Area under the curve (AUC) and 95% confidence intervals are shown

The PNC accurately classified the most prevalent NSCLC histological cell types: adenocarcinoma, AUC = 0.665 (p = 0.0001) and squamous cell carcinoma, AUC = 0.709 (p = 0.0005) from benign controls, as shown in Fig. 3. Analysis of individual biomarkers revealed that CEA and CYFRA 21-1 also distinguished adenocarcinoma (CEA: AUC = 0.665, p = 0.0004; CYFRA 21-1: AUC = 0.629, p = 0.0033) and squamous cell histologies (CEA: AUC = 0.649, p = 0.0180; CYFRA 21-1: AUC = 0.643, p = 0.0484) as shown in Additional file 1: Table S5. SCC resolved malignant squamous cell cases from benign nodules (AUC = 0.653, p = 0.0258). OPN, previously reported to be expressed at elevated levels in squamous cell carcinomas in tissue [39] and serum [40], also distinguished malignant squamous cell carcinoma cases from benign nodules (AUC = 0.664, p = 0.0039). The relatively strong performance of SCC and OPN in distinguishing squamous cell cases in the validation study is consistent with results observed in the training study (unpublished data).

As well as evaluating the diagnostic accuracy of the PNC in subjects with solitary pulmonary nodules, we also explored the performance of the classifier in individuals with other chest radiography findings considered suspicious for lung cancer (Table 2). While the PNC accurately distinguished patients with malignant masses (AUC = 0.718, p < 0.0001), it performed less well on subjects with other abnormal findings (infiltrate, hilar or pleural mass; AUC = 0.649, p = 0.0504; Additional file 2: Figure S3). The performance of individual biomarkers, including those not selected for the PNC, was also assessed in these populations (Additional file 1: Table S6). CEA (AUC = 0.689; p = 0.0004), and two of the biomarkers not included in the PNC, SPLI (AUC = 0.685; p = 0.0007) and TIMP1 (AUC = 0.670; p = 0.0019), accurately resolved malignant masses from matched benign controls (>3 cm).

## Discussion

The development of robust blood-based biomarkers to be used in the clinical setting requires thorough evaluation of pre-analytical variables, including the procedures used for blood collection and processing. Use of different blood collection tubes and variations in the time permitted for clot-formation can dramatically influence biomarker levels, potentially compromising the accuracy of test results [26, 41, 42]. In this preliminary evaluation of pre-analytical variability we examined the influence of clotting times on serum levels of candidate biomarkers in a limited number of health individuals (n = 6). Larger studies, including subjects with benign and malignant nodules are certainly warranted given the differential expression of circulating proteases in these populations [43]. Extending the time allowed for clot formation from 0.5 h to either 4 h or 24 h led to a substantial reduction in levels of MDK, prompting the removal of this biomarker from the panel of candidates. Additional studies investigating the influence or blood-collection tube-type and biomarker stability following serum separation revealed minimal levels of pre-analytical variability for the remaining 8 biomarkers candidates (unpublished data). These biomarkers were therefore included in the pool for multi-marker development.

The diagnostic accuracy of the PNC fell dramatically in the validation study (AUC = 0.653, Additional file 2: Figure S2) relative to the performance observed in the training study (AUC = 0.897, Additional file 2: Figure S1). Similar drops in multi-biomarker test performance have been reported for other tests in validation studies aimed at classifying indeterminate pulmonary nodules [14, 15]. The decline in accuracy reflects diminished performance for all 5 biomarkers that constitute the PNC, with an average fall in AUC of 0.101 (Additional file 1: Table S7). Further investigation into differences in PNC signal revealed levels in the control populations (training and validation) to be remarkably similar (Additional file 2: Figure S4). This was not the case for the malignant populations, where PNC levels observed in the validation study were substantially lower than in the training study. Some of this difference may be attributed to the reduced dimensions of the malignant lesions included in the validation study. Subjects with malignant nodules (diameter ≤3 cm) were selected for the validation study, while stage I NSCLC cases (diameter ≤5 cm) were used for training. Although the reduced dimensions of malignant lesions are likely to have played some role in the lower PNC signal in validation study, the relatively low performance of the classifier in subjects with larger pulmonary masses (diameter >3 cm; AUC = 0.718, Additional file 2: Figure S3) suggests the involvement of additional factors. It seems likely that the pre-diagnostic collection of biospecimens, together with the asymptomatic nature of the population screened, may have impacted results in the validation study.

One of the key applications for an adjunctive test to be used in the management of individuals with indeterminate pulmonary nodules is to reduce the number of unnecessary biopsies in cases with low to moderate risk of malignancy, thereby reducing the number of biopsy-related adverse events and the substantial costs associated with these procedures [10]. To achieve clinical utility in this setting, it has been suggested that an adjunctive test would need to perform with high accuracy: 80% sensitivity at 90% specificity [13]. At this level of performance, the test would reduce the number of unnecessary biopsies by approximately 80%, making it a viable alternative to FDG-PET, which is typically recommended for evaluation of this population [7]. While FDG-PET typically performs with high accuracy in distinguishing benign from malignant nodules (sensitivity = 87%, specificity = 83%) [44], it is costly, and in some settings, including regions of endemic infectious granulomatous lung disease, it’s use may be restricted [45]. With an AUC of 0.653 [95% CI 0.583–0.723] (Additional file 2: Figure S2), the PNC classifier delivered 18% sensitivity at 90% specificity, well below of the desired level of performance. It remains to be seen whether integration of clinical and radiographic variables to the biomarkers included in the PNC will result in a multi-modal test with sufficient diagnostic accuracy to achieve clinical utility. Two of the biomarkers evaluated here (CEA, CYFRA 21-1) accurately distinguish malignant pulmonary lesions from benign nodules as individual markers (Table 3) and should be considered as possible components of future multi-marker panels.

Limitations of the study include the small number of biomarkers evaluated and differences in the clinical characteristics of the populations used in training and validation. Also lacking is a demonstration of clinical utility: does the PNC actually add to the accuracy of the clinical and radiographic variables typically assessed in patient evaluation? This type of multimodal analysis [17, 18, 46–48] was not possible in the current study as some key radiographic variables, including nodule size, were not collected in the PLCO trial. A major strength of this study was the identification of candidate markers impacted by pre-analytical variables associated with sample collection and processing, and the deselection of such markers ahead of clinical validation studies. Another strength of the study stems from the utilization of samples collected in the PLCO trial to evaluate clinical accuracy. The PLCO study design not only overcomes any inherent bias in sample collection but also enables accurate selection of benign and malignant populations matched on radiographic findings and key demographic variables.

## Conclusions

As CT-screening of the population at high risk for lung cancer gains traction, the need for non-invasive markers to improve clinical decision making in asymptomatic individuals with indeterminate nodules will become ever-more pressing. The PLCO study serves as a valuable resource in the testing of candidate biomarkers for the classification of benign nodules from malignant lesions in the setting of radiographic screening for lung cancer. Our study provides valuable insight into the clinical accuracy of a pulmonary nodule classifier and a number of well characterized biomarkers: CEA, CYFRA 21-1 and SCC, in the asymptomatic population. As well as evaluating diagnostic performance in individuals with pulmonary nodules, these biomarkers have also been characterized in subjects with other commonly encountered radiological findings. Some of the biomarkers included in the PNC should be further evaluated as viable complementing components of future blood-based adjunctive tests to be used in the diagnostic assessment of asymptomatic subjects with pulmonary nodules.

## Additional files

**Additional file 1.** Supplementary Tables S1 - S7

**Additional file 2.** Supplementary Figures S1 - S4

**Additional file 3.** Preanalytical Data

**Additional file 4.** Training Data

**Additional file 5.** Validation Data

## Abbreviations

AUC: area under curve
CT: computed tomography
CEA: carcinoembryonic antigen-related cell adhesion molecule 5
CMS: Centers for Medicare & Medicaid Services
ctDNA: circulating tumor DNA
CYFRA 21-1: cytokeratin-19 fragment
ELISA: enzyme-linked immunosorbent assay
FDG-PET: fluoro deoxy glucose-positron emission tomography
MDK: midkine
MMP2: matrix metalloproteinase-2
NLST: National Lung Screening Trial
NPV: negative predictive value
NSCLC: non-small cell lung cancer
OPN: osteopontin
PLCO: Prostate, Lung, Chest and Ovarian
PNC: pulmonary nodule classifier
PPV: positive predictive value
SCC: squamous cell carcinoma antigen
TFPI: tissue factor pathway inhibitor
TIMP1: tissue inhibitor of metalloproteinases-1
USPSTF: US Preventive Services Task Force
