# Supplementary material for: Clinical validation of a blood-based classifier for diagnostic evaluation of asymptomatic individuals with pulmonary nodules
Source: Clin Proteomics. 2017 Jul 5;14:25. doi: 10.1186/s12014-017-9158-9 (PMC5498919; doi:10.1186/s12014-017-9158-9)
Supplement: Supplementary file 1 — Additional file 1. Supplementary Tables S1 - S7 [file 12014_2017_9158_MOESM1_ESM.docx]

| **Additional file 1: Table S1.** Reagents used in study | |  |  |
| --- | --- | --- | --- |
|  | | **Source** | **Catalog #** |
| **Biomarker** | |  |  |
| h CYFRA 21-1 | | SunnyLabs | P246-4 |
| rh Serpin B3 (SCCA1) | | R&D Systems (Bio-Techne) | 6528-PI |
| rh CEACAM-5 (CEA) | | R&D Systems (Bio-Techne) | 4128-CM |
| rh Osteopontin (OPN) | | R&D Systems (Bio-Techne) | 1433-OP/CF |
| rh TFPI | | R&D Systems (Bio-Techne) | 2974-PI |
| rh SLPI | | R&D Systems (Bio-Techne) | 1274-PI |
| rh TIMP-1 | | R&D Systems (Bio-Techne) | 970-TM |
| rh MDK | | R&D Systems (Bio-Techne) | 258-MD |
| rh MMP-2 | | R&D Systems (Bio-Techne) | 902-MP |
| **Primary (capture) antibody** | Bead Conjugated (Y/N) |  |  |
| α-CEA Beads | Y | Millipore | HCEA-MAG |
| α -MMP-2 Beads | Y | R&D Systems (Bio-Techne) | LMPM902 |
| Mu- α -CYFRA, # KS19.21 | N | Fujirebio | 206-575 |
| Mu- α -SCC, # SCC140 | N | Fujirebio | 805-01 |
| Mu- α -OPN | N | R&D Systems (Bio-Techne) | MAB14332 |
| Mu- α -TFPI | N | R&D Systems (Bio-Techne) | MAB29741 |
| Mu- α -SLPI | N | R&D Systems (Bio-Techne) | MAB1274 |
| Mu- α -TIMP-1 | N | R&D Systems (Bio-Techne) | MAB970 |
| Mu- α -MDK, # IP10 | N | Abcam | ab52318 |
| **Secondary (detection) antibody** | Biotinylated (Y/N) |  |  |
| Gt- α -OPN | Y | R&D Systems (Bio-Techne) | BAF1433 |
| Shp- α CEA | Y | R&D Systems (Bio-Techne) | BAF4128 |
| Gt- α -TFPI | Y | R&D Systems (Bio-Techne) | BAF2974 |
| Gt- α -SLPI | Y | R&D Systems (Bio-Techne) | BAF1274 |
| Gt- α -TIMP-1 | Y | R&D Systems (Bio-Techne) | BAF970 |
| Gt- α -MDK | Y | R&D Systems (Bio-Techne) | BAF258 |
| Gt- α -MMP-2 | Y | R&D Systems (Bio-Techne) | BAF902 |
| Mu- α -CYFRA, # BM19.21 | N | Fujirebio | 206-580 |
| Mu- α -SCC, # SCC107 | N | Fujirebio | 800-25 |

**Additional file 1: Table S2.** Multiplex Cross Reactivity

Multiplex 1: Undiluted Serum

|  | CEA | CYFRA 21-1 | SCC | MDK |
| --- | --- | --- | --- | --- |
| CEA | 99.2% | 0.00% | 0.07% | -0.03% |
| CYFRA 21-1 | 0.42% | 101.8% | 0.09% | 0.74% |
| SCC | 0.00% | 0.02% | 99.7% | 0.64% |
| MDK | 0.01% | 0.04% | 0.17% | 101.8% |

Multiplex 2: Serum diluted 1:10

|  | MMP2 | OPN | SLPI | TFPI | TIMP1 |
| --- | --- | --- | --- | --- | --- |
| MMP2 | 107.2% | -0.02% | 0.00% | -0.39% | 0.00% |
| OPN | 0.02% | 104.2% | 0.00% | 0.86% | 0.01% |
| SLPI | 0.03% | 0.01% | 101.3% | 0.51% | 0.01% |
| TFPI | 0.05% | 0.00% | 0.10% | 99.3% | 0.06% |
| TIMP1 | 0.00% | -0.03% | 0.02% | -0.38% | 100.1% |

The multiplex assay is highly specific with <1% cross-reactivity among markers. Measurements were calculated from the third highest concentration in standard curves of three-fold serial dilution of individual and multiplexed markers [1]. Cross-reactivity was determined by measuring bead signal (mean fluorescence intensity) from a single analyte with multiplexed capture beads and multiplexed detection antibodies, relative to the bead signal observed with the full multiplex assay (all analytes, all capture beads, all detection antibodies).

| **Additional file 1: Table S3.** Diagnostic accuracy of candidate markers in the training study | | |
| --- | --- | --- |
|  | Training | |
|  | Control (n = 186); NSCLC (n = 95) | |
|  | AUC [95% CI] | p value |
|  |  |  |
| *Individual markers included in PNC* | | |
| CEA | 0.717 [0.651 - 0.783] | <0.0001 |
| CYFRA 21-1 | 0.721 [0.653 - 0.789] | <0.0001 |
| SCC | 0.686 [0.610 - 0.762] | <0.0001 |
| OPN | 0.712 [0.645 - 0.779] | <0.0001 |
| TFPI | 0.572 [0.497 - 0.647] | 0.0588 |
|  |  |  |
| *Individual markers not included in PNC* |  |  |
| MMP2 | 0.548 [0.468 - 0.627] | 0.2406 |
| SLPI | 0.550 [0.472 - 0.627] | 0.2102 |
| TIMP1 | 0.517 [0.442 - 0.592] | 0.6519 |
|  | | |

| **Additional file 1: Table S4.** Diagnostic accuracy of Pulmonary Nodule Classifier (PNC) and individual markers in the nodule population (validation study), relative to tumor stage | | | | | | | | | | | | | | | | | | |
| --- | --- | --- | --- | --- | --- | --- | --- | --- | --- | --- | --- | --- | --- | --- | --- | --- | --- | --- |
|  |  | | | Validation  CXR Finding: Nodule | | | | | | | | | | | | | | |
|  | Stage IA  (n = 51) | | | Stage IB  (n = 15) | | |  | Stage II  (n = 10) | | |  | Stage III  (n = 19) | |  | Stage IV  (n = 14) | | | |
|  | AUC p value | | | AUC | p value | |  | AUC | | p value |  | AUC | p value |  | AUC | | p value | |
| PNC | 0.618 0.0071 | | | 0.557 | 0.4942 | |  | 0.695 | | 0.0341 |  | 0.766 | <0.0001 |  | 0.742 | | 0.0009 | |
|  |  | | |  |  | |  |  | |  |  |  |  |  |  | |  | |
| *Individual markers included in PNC* | | | | | | | | | | | | | | | | | | |
| CEA | | 0.573 0.1352 | | 0.631 | | 0.1100 |  | 0.850 | <0.0001 | |  | 0.683 | 0.0175 |  | 0.687 | | | 0.0462 |
| CYFRA 21-1 | | 0.578 0.0955 | | 0.451 | | 0.6400 |  | 0.692 | 0.0517 | |  | 0.765 | <0.0001 |  | 0.734 | | | 0.0012 |
| SCC | | 0.561 0.2172 | | 0.532 | | 0.7051 |  | 0.480 | 0.8326 | |  | 0.586 | 0.2348 |  | 0.658 | | | 0.0525 |
| OPN | | 0.564 0.1843 | | 0.489 | | 0.8927 |  | 0.503 | 0.9777 | |  | 0.617 | 0.0931 |  | 0.462 | | | 0.6146 |
| TFPI | | 0.531 0.5199 | | 0.507 | | 0.9250 |  | 0.414 | 0.4886 | |  | 0.569 | 0.3617 |  | 0.568 | | | 0.3828 |
|  | |  | |  | |  |  |  |  | |  |  |  |  | |  | |  |
|  | | |  | | | | | | | | | | | | | | | |

Accuracy of the PNC and individual markers for differentiation of control patients with benign nodules (n= 119) from cancer patients was determined relative to tumor stage.

| **Additional file 1: Table S5.** Diagnostic accuracy of Pulmonary Nodule Classifier (PNC) and individual markers in the nodule population (validation study), relative to tumor histology | | | | | | | | | | |
| --- | --- | --- | --- | --- | --- | --- | --- | --- | --- | --- |
|  |  | Validation  CXR Finding: Nodule | | | | | | | | |
|  |  | Adenocarcinoma (n = 57) | | |  | Squamous cell  (n = 25) | |  | Other  (n=37) | |
|  |  | AUC | p value | |  | AUC | p value |  | AUC | p value |
| PNC |  | 0.665 | 0.0001 | |  | 0.709 | 0.0005 |  | 0.596 | 0.0628 |
|  |  |  |  | |  |  |  |  |  |  |
| *Individual markers included in PNC* | | | | | | | | | | |
| CEA |  | 0.665 | 0.0004 |  | | 0.649 | 0.0180 |  | 0.602 | 0.0665 |
| CYFRA 21-1 |  | 0.629 | 0.0033 |  | | 0.643 | 0.0484 |  | 0.617 | 0.0327 |
| SCC |  | 0.552 | 0.2790 |  | | 0.653 | 0.0258 |  | 0.531 | 0.5621 |
| OPN |  | 0.525 | 0.5905 |  | | 0.664 | 0.0039 |  | 0.464 | 0.5063 |
| TFPI |  | 0.523 | 0.6270 |  | | 0.592 | 0.1352 |  | 0.508 | 0.8845 |

Accuracy of the PNC and individual markers for differentiation of control patients with benign nodules (n = 119) from cancer patients was determined relative to tumor histology.

| **Additional file 1: Table S6.** Diagnostic accuracy of Pulmonary Nodule Classifier (PNC) and individual markers evaluated in validation study populations: masses, other findings | | | | | |
| --- | --- | --- | --- | --- | --- |
|  | Validation | | | | |
|  | CXR Finding: Mass  Benign Control (n = 50),  Lung Cancer (n = 50) | |  | CXR Finding: Other  Benign Control (n = 28),  Lung Cancer (n = 28) | |
|  | AUC | p value |  | AUC | p value |
| PNC | 0.718 | <0.0001 |  | 0.649 | 0.0504 |
|  |  |  |  |  |  |
| *Individual markers included in PNC* | | | | | |
| CEA | 0.689 | 0.0004 |  | 0.686 | 0.0149 |
| CYFRA 21-1 | 0.611 | 0.0506 |  | 0.603 | 0.1796 |
| SCC | 0.519 | 0.7492 |  | 0.546 | 0.5595 |
| OPN | 0.592 | 0.1100 |  | 0.517 | 0.8353 |
| TFPI | 0.532 | 0.5896 |  | 0.467 | 0.6728 |
|  |  |  |  |  |  |
| *Individual markers not included in PNC* | | | | | |
| MMP2 | 0.510 | 0.8703 |  | 0.575 | 0.3350 |
| SLPI | 0.685 | 0.0007 |  | 0.596 | 0.2176 |
| TIMP1 | 0.670 | 0.0019 |  | 0.608 | 0.1604 |

| **Additional file 1: Table S7.** Diagnostic Accuracy (AUC) of PNC and Individual Markers in Training and Validation Studies | | | |
| --- | --- | --- | --- |
|  | Training  Study  (AUC) | Validation  Study  (AUC) | Δ Studies  (Validation – Training)  (AUC) |
| PNC | 0.897 | 0.653 | -0.244 |
| *Individual markers included in PNC* | | | |
| CEA | 0.717 | 0.642 | -0.075 |
| CYFRA 21-1 | 0.721 | 0.628 | -0.093 |
| SCC | 0.686 | 0.567 | -0.119 |
| OPN | 0.712 | 0.535 | -0.177 |
| TFPI | 0.572 | 0.533 | -0.039 |
| 5 marker (mean) | 0.682 | 0.581 | -0.101 |

References

1. Angeloni S, Cordes R, Dunbar S, Garcia C, Gibson G, Martin C, Stone V: **xMAP® Cookbook: A collection of methods and protocols for developing multiplex assays with xMAP Technology.** *Luminex* 2016.
