# Supplementary material for: Clinical validation of a blood-based classifier for diagnostic evaluation of asymptomatic individuals with pulmonary nodules
Source: Clin Proteomics. 2017 Jul 5;14:25. doi: 10.1186/s12014-017-9158-9 (PMC5498919; doi:10.1186/s12014-017-9158-9)
Supplement: Supplementary file 2 — Additional file 2. Supplementary Figures S1 - S4 [file 12014_2017_9158_MOESM2_ESM.pptx]

## Slide 1
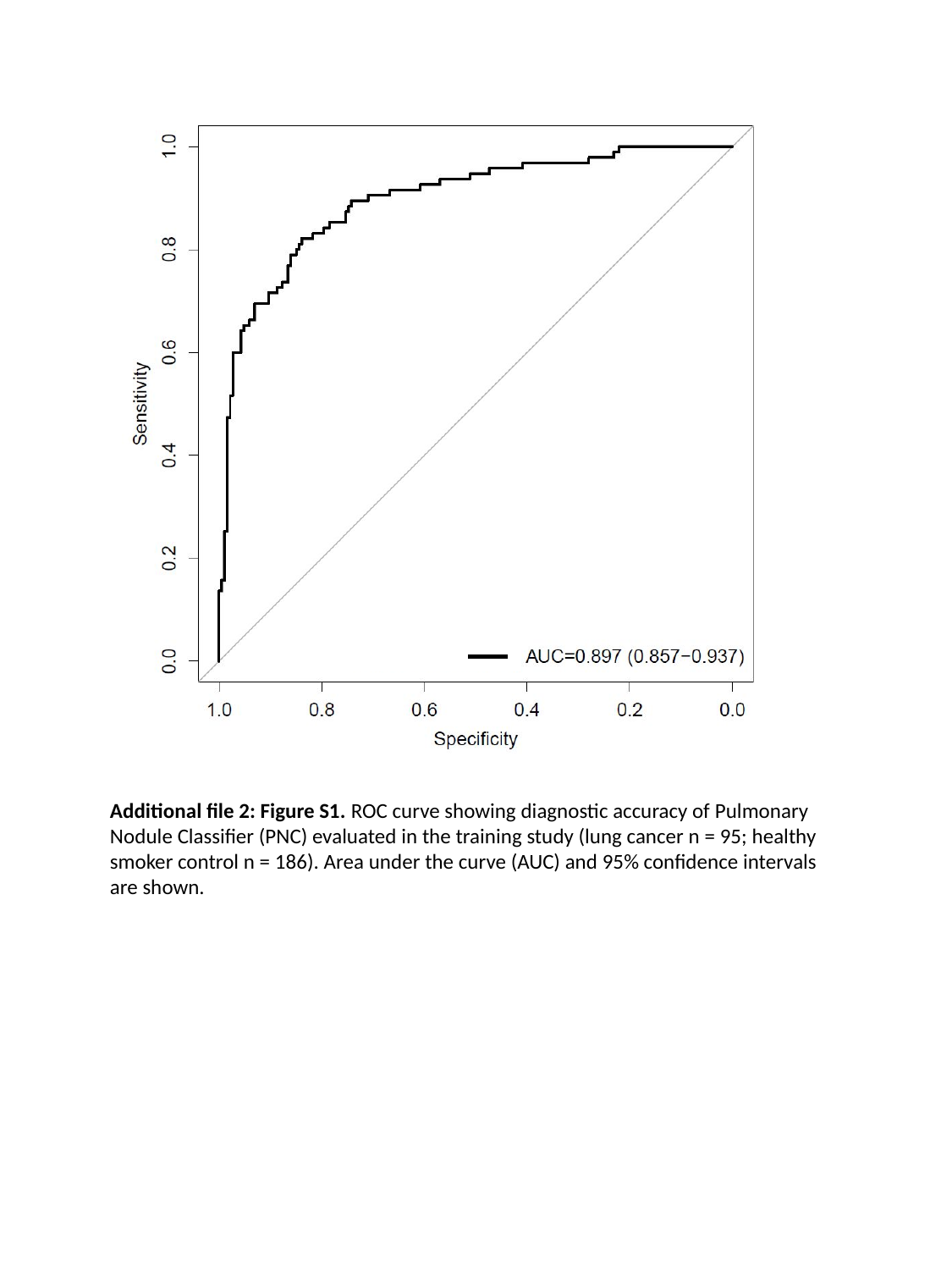

Additional file 2: Figure S1. ROC curve showing diagnostic accuracy of Pulmonary Nodule Classifier (PNC) evaluated in the training study (lung cancer n = 95; healthy smoker control n = 186). Area under the curve (AUC) and 95% confidence intervals are shown.

## Slide 2
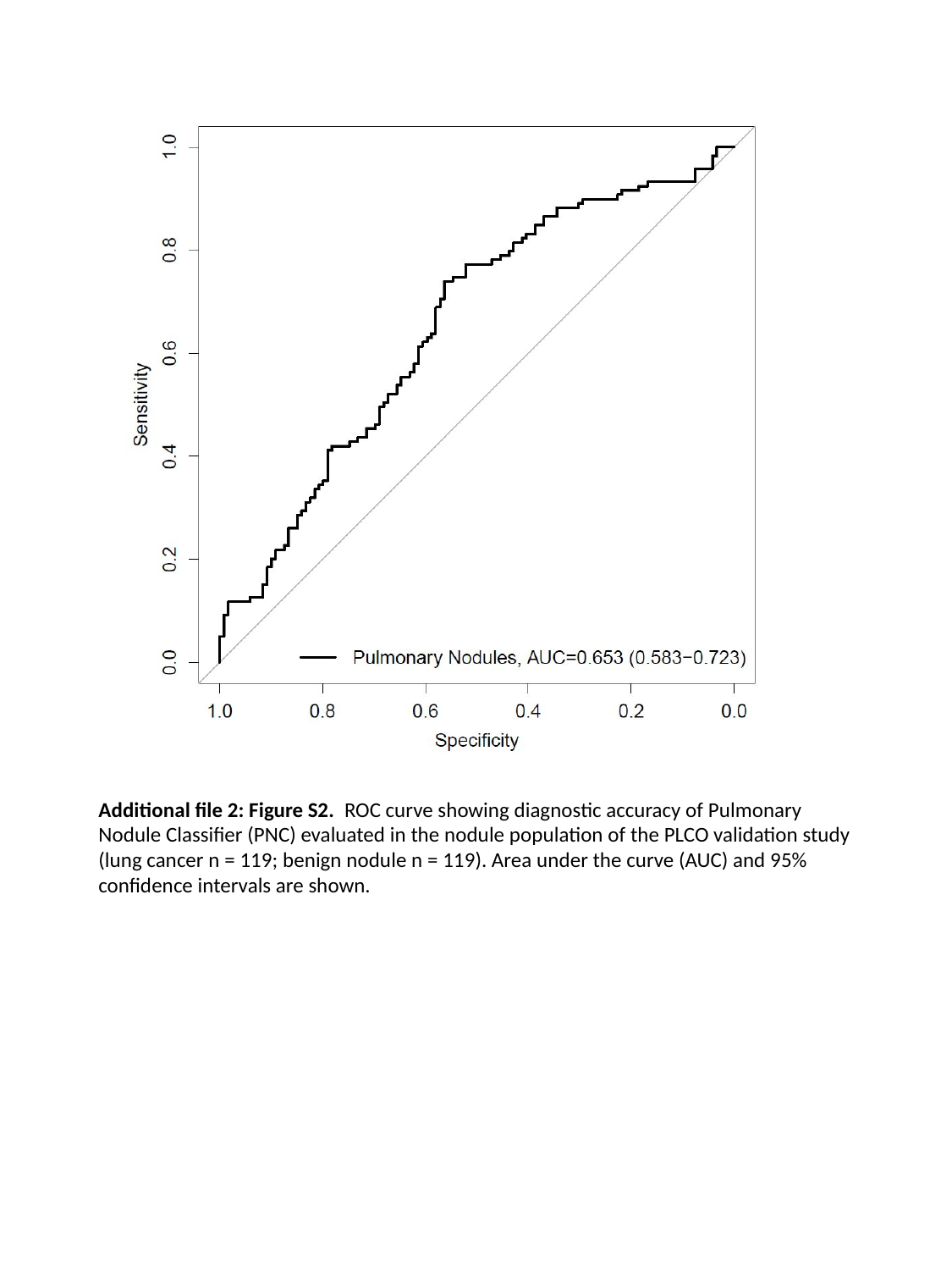

Additional file 2: Figure S2. ROC curve showing diagnostic accuracy of Pulmonary Nodule Classifier (PNC) evaluated in the nodule population of the PLCO validation study (lung cancer n = 119; benign nodule n = 119). Area under the curve (AUC) and 95% confidence intervals are shown.

## Slide 3
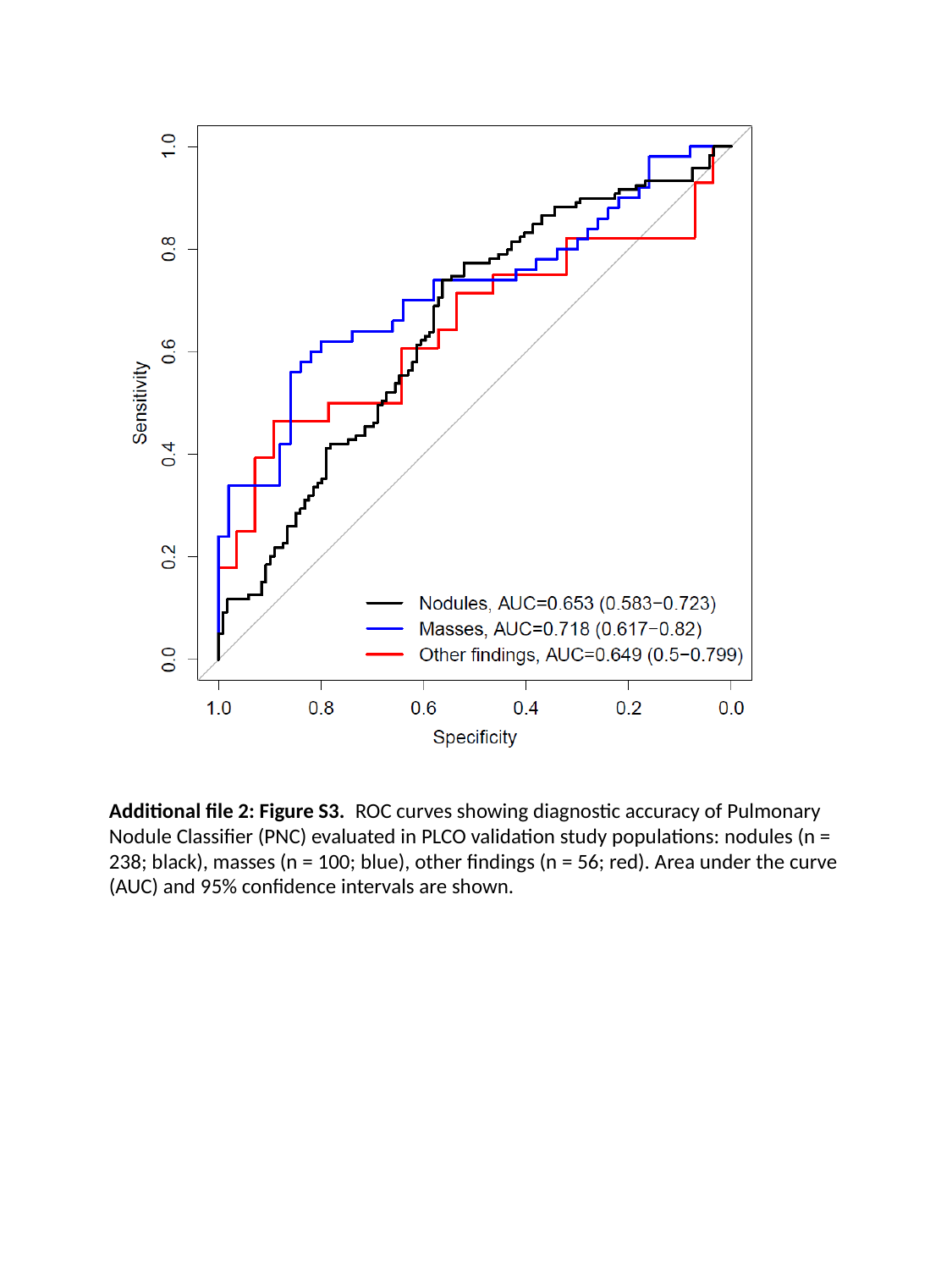

Additional file 2: Figure S3. ROC curves showing diagnostic accuracy of Pulmonary Nodule Classifier (PNC) evaluated in PLCO validation study populations: nodules (n = 238; black), masses (n = 100; blue), other findings (n = 56; red). Area under the curve (AUC) and 95% confidence intervals are shown.

## Slide 4
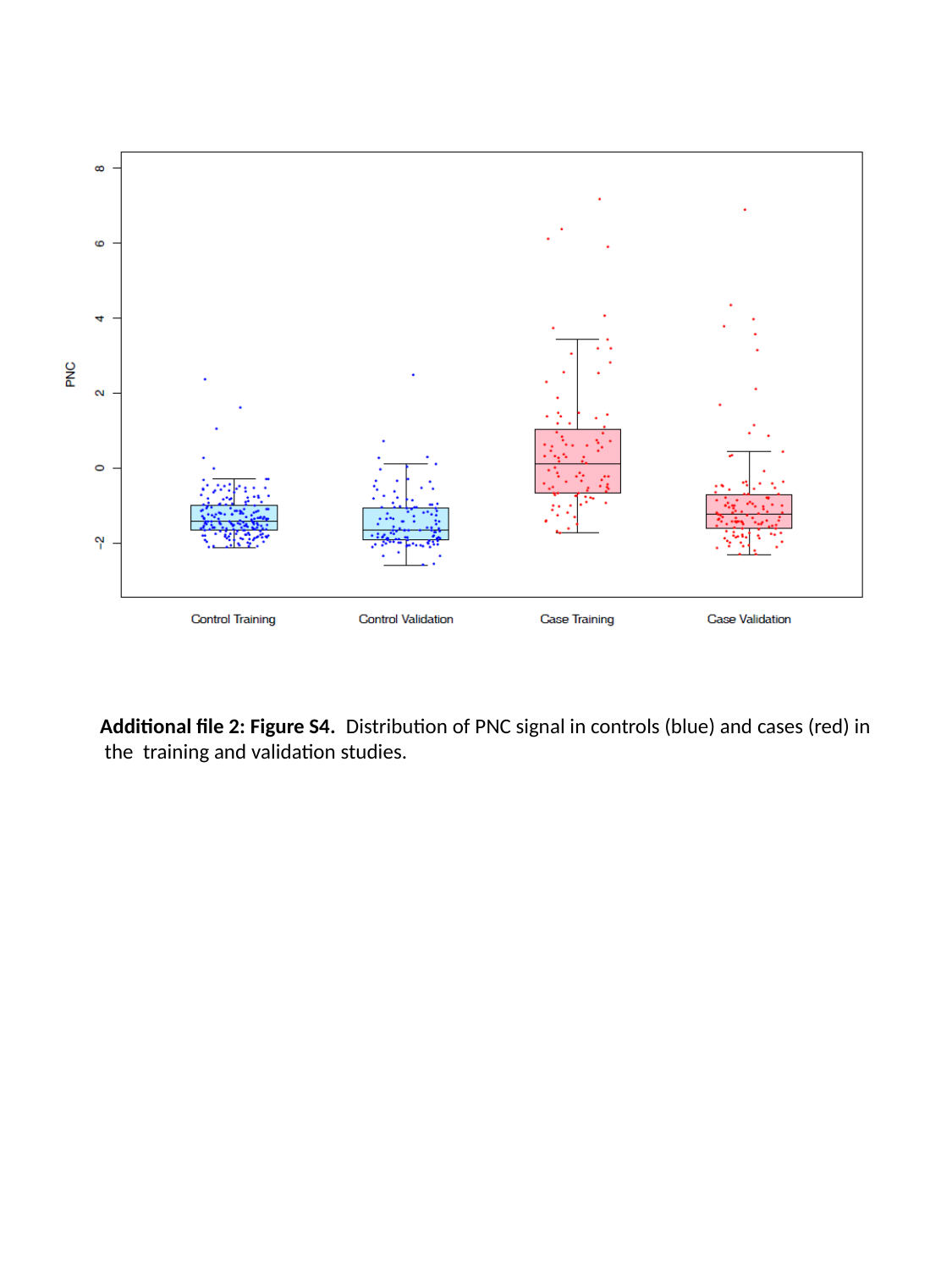

Additional file 2: Figure S4. Distribution of PNC signal in controls (blue) and cases (red) in the training and validation studies.
